# Functional alterations of the brain default mode network and somatosensory system in trigeminal neuralgia

**DOI:** 10.1038/s41598-024-60273-6

**Published:** 2024-05-03

**Authors:** Zairan Wang, Zijun Zhao, Zihan Song, Jiayi Xu, Yizheng Wang, Zongmao Zhao, Yongning Li

**Affiliations:** 1 Department of Neurosurgery, Peking Union Medical College Hospital, Chinese Academy of Medical Sciences, No.1 Shuaifuyuan Wangfujing, Dongcheng District, Beijing, China; 2 Spine Center, Sanbo Brain Hospital, Capital Medical University, Beijing, China; 3 Department of Neurosurgery, The Second Hospital of Hebei Medical University, Shijiazhuang, Hebei China; 4 Medical Records Room, The First Hospital of Hebei Medical University, Shijiazhuang, Hebei China; 5 Department of Pain Rehabilitation, The Forth Hospital of Hebei Medical University, Shijiazhuang, Hebei China; 6 Department of Neurosurgery, The Fourth Hospital of Hebei Medical University, Shijiazhuang, Hebei China

**Keywords:** Trigeminal neuralgia, Brain mapping, Functional neuroimaging, Default mode network, Somatosensory disorders, Pain, Neuroscience

## Abstract

Mapping the localization of the functional brain regions in trigeminal neuralgia (TN) patients is still lacking. The study aimed to explore the functional brain alterations and influencing factors in TN patients using functional brain imaging techniques. All participants underwent functional brain imaging to collect resting-state brain activity. The significant differences in regional homogeneity (ReHo) and amplitude of low frequency (ALFF) between the TN and control groups were calculated. After familywise error (FWE) correction, the differential brain regions in ReHo values between the two groups were mainly located in bilateral middle frontal gyrus, bilateral inferior cerebellum, right superior orbital frontal gyrus, right postcentral gyrus, left inferior temporal gyrus, left middle temporal gyrus, and left gyrus rectus. The differential brain regions in ALFF values between the two groups were mainly located in the left triangular inferior frontal gyrus, left supplementary motor area, right supramarginal gyrus, and right middle frontal gyrus. With the functional impairment of the central pain area, the active areas controlling memory and emotion also change during the progression of TN. There may be different central mechanisms in TN patients of different sexes, affected sides, and degrees of nerve damage. The exact central mechanisms remain to be elucidated.

## Introduction

Trigeminal neuralgia (TN) is a chronic brain disease involving the trigeminal nerve^1^. Neurovascular compression (NVC) in the root entry zone of the brainstem and the excitability of the nerve fibers involved are the potential causes^2,3^. However, NVC of the trigeminal nerve is present on both the asymptomatic and symptomatic sides in most TN patients^4^. Many TN patients with substantial NVC fail to achieve long-term pain relief after technically successful surgery^5,6^. In this way, the peripheral mechanisms alone do not adequately explain the onset of TN. It is, therefore, essential to explore the central mechanisms of TN. Mapping the localization of the functional brain regions in TN patients is still lacking.

Resting-state functional magnetic resonance imaging (rs-fMRI) is a suitable technique for studying central mechanisms. It enables direct observation of subjects’ spontaneous neurological brain activity at rest without other activity, emphasizing the parallelism of the human neural network and the significance of the relationships between brain regions^7^. The amplitude of low frequency (ALFF) is a valuable metric based on rs-fMRI that can be used to assess the intrinsic fluctuations of the signal to reflect the intensity of spontaneous neural activity^8^. Changes in ALFF values are often thought to be related to metabolic levels^9^. Regional homogeneity (ReHo) was applied to investigate the coherence of spontaneous neuronal activity between a given voxel and its neighbors at rest, further clarifying differences in local brain activity. Changes in ReHo values reveal possible abnormalities in the synchronization and coordination of spontaneous neuronal activity. Thus, ALFF and ReHo have become practical tools for measuring brain structure and better understanding pathophysiological changes in brain function.

The default mode network (DMN) is the most stable and well-studied resting-state brain network. It is generally divided into three subsystems: medial prefrontal region, lateral parietal cortex and precuneus regions, and medial parietal and posterior cingulate regions. In addition to functions such as memory and cognition, the DMN is involved in various cognitive tasks, including those unrelated to task stimuli^9–11^. It is usually active when not in contact with the external environment^12^. The DMN is involved in mediating pain feedback. In turn, Potential alterations in DMN activity may be related to symptoms (in addition to pain) often exhibited by people with chronic pain, including anxiety, depression, sleep disturbances, and abnormal decision-making^13^, which greatly reduce their quality of life. Neuropathic pain is a common form of chronic pain caused by lesions or diseases of the somatosensory nervous system^14^. Accumulating evidence suggests that the brain’s DMN and somatosensory systems play a vital role in the pathophysiology of chronic pain in humans^9,15–20^. The study aimed to explore the functional brain alterations and influencing factors in TN patients using functional brain imaging techniques.

## Materials and methods

### Ethics statement

The study was approved (2022-R281) by the Ethics Committee of the Second Hospital of Hebei Medical University, Hebei, China, and subjects signed informed consent to participate in the study. The study was carried out in accordance with the Declaration of Helsinki.

### Participants

Thirty-eight TN patients (TN group; 11 males and 27 females; age 62.2 ± 10.3 years) and 20 healthy controls (control group; 8 males and 13 females; age 56.8 ± 9.4 years) were included in this study. Inclusion criteria were (i) unilateral pain involving one or more branches of the trigeminal nerve; (ii) paroxysmal facial pain lasting from seconds to minutes; (iii) sharp, burning or piercing pain with characteristic trigger points or trigger factors; (iv) routine magnetic resonance imaging (MRI) examination showing no obvious abnormal brain signal; (v) no obvious clinical neurological or sensory dysfunction; (vi) duration of more than six months (chronic pain patients) and (vii) right-handed. The diagnosis of TN was determined according to the International Classification of Headache Disorders (3rd Edition)^1^. The exclusion criteria were (i) migraine or other paroxysmal or chronic pain; (ii) secondary TN caused by intracranial tumors, multiple sclerosis or other diseases; (iii) neurological or psychiatric diseases, including traumatic brain injury, brain tumors, cerebral hemorrhage and other neurological and psychiatric conditions; (iv) history of alcohol or drug abuse; and (v) contraindications to magnetic resonance scanning.

### Pain evaluation

An evaluation of TN pain was conducted prior to MRI scan using the visual analog scale (VAS). By using a 10 cm ruler, patients rated their pain from 0 to 10. An evaluation of 0 means no pain, and a rating of 10 means severe, extremely intolerable pain^21^.

### MRI data collection

For the MRI scan, a Philips Achieva 3.0 T X-series MRI machine was used for scanning with an eight-channel phase array head coil. Participants were instructed to close their eyes, remain awake, and breathe quietly until the scan was complete. The scanning sequence included three-dimensional fast imaging (3D-DRIVE), high resolution (HR) 3D T1 weighted imaging (T1WI), and rs-fMRI. The parameters of the 3D-DRIVE were set as follows: repetition time (TR)/echo time (TE) 1500/200 ms; field of view (FOV) 200 mm × 200 mm; slice thickness 0.5 mm; slice spacing 0 mm; matrix 640 × 640. The scanning baseline was parallel to the auditory-orbital line and extended from the frontal base to the bridging sulcus, including the trigeminal nerve, facial auditory nerve, and the main branches of the vertebrobasilar artery. The 3D-DRIVE sequence was reconstructed in the oblique sagittal and coronal positions. HR structural images were collected using a sagittal fast field echo (FFE) sequence with the following parameters: TR/TE 7.61/3.71 ms, number of excitations (NEX) 1, flip angle (FA) 8°, FOV 240 mm × 240 mm, matrix 240 × 240, voxel 1 mm × 1 mm, slice thickness 1 mm, and slice spacing 0 mm. Gradient-recalled echo (GRE) echo-planar imaging (EPI) sequences were used to collect functional data. The parameters were as follows: TR/TE 2000/30 ms, NEX 1, FA 90°, FOV 240 mm × 240 mm, acquisition matrix 64 × 64, voxel 3.75 mm × 3.75 mm × 4.0 mm, number of plies 35, slice thickness 4.0 mm, slice spacing 0 mm, Each scanning process lasted 8 min; and 240 time points were collected. Under the premise of constant acquisition time, parallel acquisition technology is adopted to reduce magnetic sensitive artifacts.

### Evaluation of NVC grading quantitatively and qualitatively

Two senior radiologists blinded to the symptomatic side analyzed the MRI features of all neurovascular contacts of the bilateral trigeminal nerves in all enrolled subjects. Transverse, coronal, and sagittal planes were used to evaluate the relationship between the blood vessels and the trigeminal nerve according to previous literature assessment criteria^22^. The scoring criteria for NVC degree were as follows: Null point without contact; 1 point contact between nerves and blood vessels with no indentation or nerve shift; 2 points contact between nerves and blood vessels with indentation; and 3 points nerve compression due to blood vessels, resulting in distortion or displacement of nerves. A 9-point scale was used to grade nerve compression by blood vessels (NVC grading), which sums up the NVC points on transverse, sagittal, and oblique coronal planes.

### Image processing

DPARSF v2.0 software based on MATLAB R2012a was used for preprocessing. Processing steps included slice timing, head-motion correction, and spatial normalization in the Montreal Neurological Institute (MNI) space. The linear trend of the fMRI data was removed. For ReHo, Kendall’s coefficient concordance (KCC) was used as an indicator to measure consistency by collecting a given voxel and those of its neighbors (26 voxels) in the resting state time series. We used Gaussian random field (GRF) theory and recorded average ReHo values in different brain regions between the two groups. On the basis of preprocessing, four-dimensional resting fMRI data were obtained by low-frequency filtering, and their time series were converted to frequency bands by a fast Fourier transform. Then, the frequency band filtering was averaged at 0.01 to 0.08 Hz, and Fisher Z-transform was performed to obtain the individual Z-transform ALFF. Two independent sample t tests were used to compare voxels, with age as a covariate.

### Statistical analysis

The demographic and clinical variables of the TN and control groups were assessed using SPSS 21.0 software and an independent sample t test. *P* < 0.05 was considered to be statistically significant. The DPABI brain function data package (http://wiki.rfmri.org) was used to conduct two independent samples t tests for the above data, and the default mask was used in the test process^23^. Finally, the differential maps of ReHo and ALFF in the two groups of brain activation regions were obtained, and the number of MNI coordinates, anatomical positioning and activation intensity (T value) of the different brain regions were recorded and analyzed. We performed ALFF and ReHo analyses on both data sets with voxel level familywise error (FWE) correction (*P* < 0.01) and cluster level (*P* < 0.001). A threshold of > 18 voxels was set by AlphaSim program.

## Results

### Demographic and clinical characteristics

Table 1 shows the demographics and characteristics of the TN and control groups. There was no significant difference in demographic variables (sex, age, and education) between the two groups (*P* > 0.05). The VAS scores, duration of illness and NVC score in the TN group were 8.32 ± 1.00, 5.23 ± 6.18 years and 4.45 ± 2.85, respectively.

**Table 1:** Demographic and clinical characteristics of the TN and control groups.

	TN (n = 38)	Controls (n = 20)	P-Value
Age (years)	62.2 ± 10.3	56.8 ± 9.4	0.069 (2-simple t test)
Sex (male/female)	11/27	8/12	0.61 (Chi-squared test)
Education (years)	9.29 ± 3.47	10.00 ± 3.00	0.46 (2-simple t test)
Illness localization (left/right)	18/20	NA	NA
VAS score	8.32 ± 1.00	NA	NA
Duration of illness (years)	5.23 ± 6.18	NA	NA
NVC score	4.45 ± 2.85	NA	NA

Values are presented as mean ± standard deviation unless otherwise indicated.

*NA* not applicable.

### Brain areas with ReHo and ALFF differences between the TN and control groups

**Figure 1:**
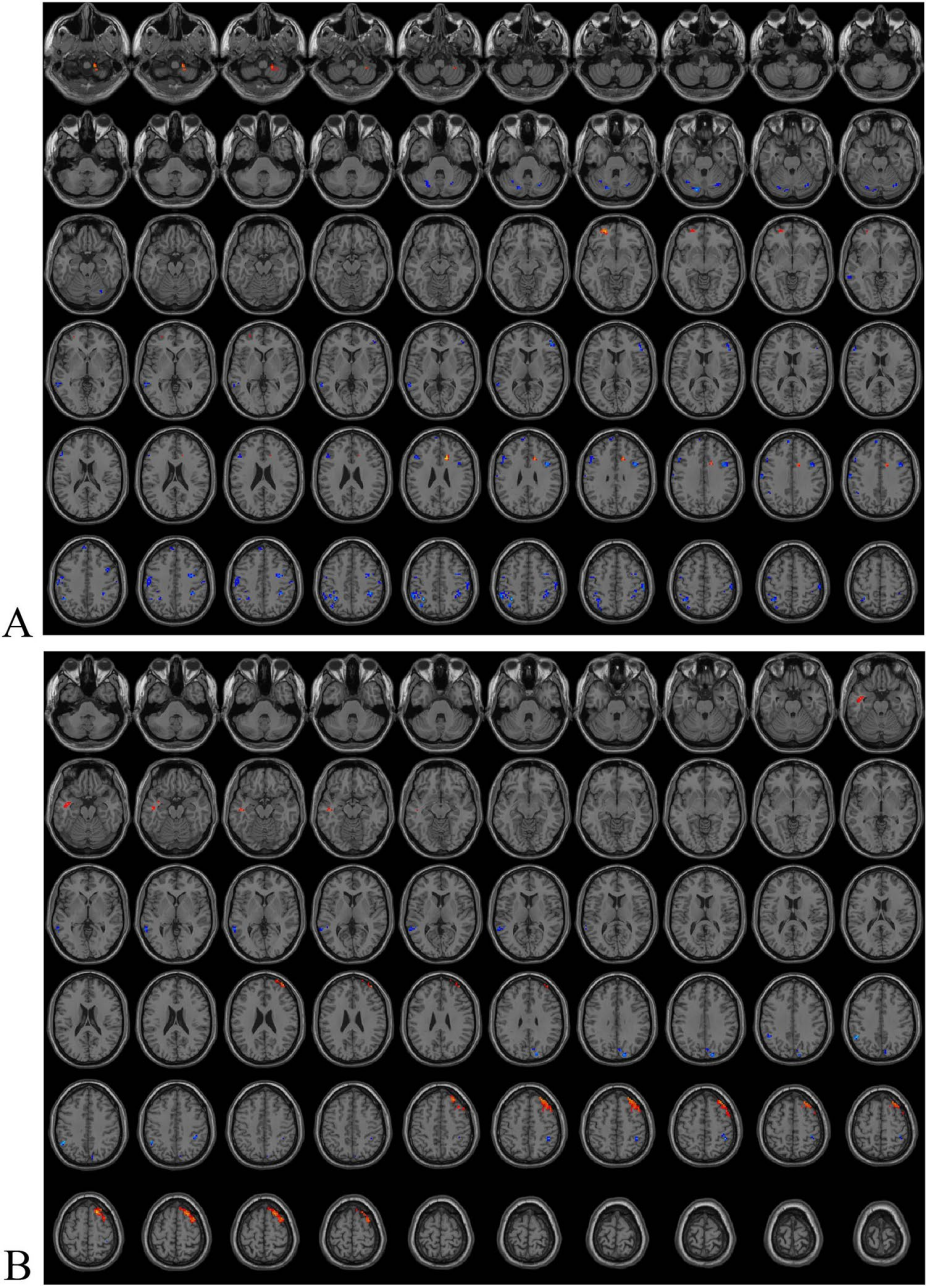
Brain areas with ReHo (**A**) and ALFF (**B**) differences between TN and Control groups.

Compared to controls, brain regions with significantly enhanced ReHo values in TN included bilateral inferior cerebellum, right superior orbital frontal gyrus, right precentral gyrus, left superior cerebellum, left gyrus rectus, and left middle temporal gyrus (Fig. 1A, Table 2). Compared to controls, brain regions with significantly diminished ReHo values in the TN group included right median cingulate gyrus, right middle frontal gyrus, right supramarginal gyrus, left triangular inferior frontal gyrus, left middle temporal gyrus, and left middle frontal gyrus (Fig. 1A, Table 2). After FWE correction, the differential brain regions in ReHo values between the two groups were mainly located in the bilateral middle frontal gyrus, bilateral inferior cerebellum, right superior orbital frontal gyrus, right postcentral gyrus, left inferior temporal gyrus, left middle temporal gyrus, and left gyrus rectus (Table 2). Compared to controls, brain regions with significantly enhanced ALFF values in the TN group in right postcentral gyrus (Fig. 1B, Table 2). Compared to controls, brain regions with significantly diminished ALFF values in the TN group included left triangular inferior frontal gyrus, left supplementary motor area, left anterior cingulate, right supramarginal gyrus, and right middle frontal gyrus (Fig. 1B, Table 2). After FWE correction, the differential brain regions in ALFF values between the two groups were mainly located in the left triangular inferior frontal gyrus, left supplementary motor area, right supramarginal gyrus, and right middle frontal gyrus (Table 2).

**Table 2:** Diferences in ReHo and ALFF values between different groups.

Brain areas	Peak MNI coordinate			Voxel (n)	T Value
	X	Y	Z		
ReHo					
TN vs. control					
Middle frontal gyrus_L*	− 27	6	54	43	− 4.808
Middle temporal gyrus_L*	− 54	3	− 18	21	− 4.1403
Middle frontal gyrus_R*	36	27	39	46	− 4.1141
Middle temporal gyrus_L*	− 48	− 48	15	138	− 3.9814
Inferior frontal gyrus (triangular part)_L	-48	15	6	20	− 3.7488
Supramarginal gyrus_R	60	-39	33	34	− 3.73
Inferior frontal gyrus (triangular part)_L	-42	18	30	18	− 3.4864
Median cingulate gyri_R	6	24	30	19	− 3.2867
Precentral gyrus_R	57	0	48	29	3.3917
Cerebelum_Crus1_L	− 51	− 48	− 39	50	3.4893
Rectus_L*	− 3	39	− 24	109	4.0657
Cerebelum_Crus2_L*	− 45	− 69	− 45	136	4.4417
Superior frontal gyrus (orbital part)_R*	6	60	− 21	59	4.6185
Cerebelum_Crus2_R*	39	− 78	− 48	127	4.6275
Middle temporal gyrus_L*	− 63	− 15	− 24	49	4.6667
Inferior temporal gyrus_L*	− 42	6	− 33	10^#^	− 4.3179
Middle temporal gyrus_L*	− 57	− 60	21	25^#^	− 3.9536
Postcentral gyrus_R*	66	0	27	9^#^	3.982
Left vs. right affected side					
Middle frontal gyrus_R*	33	27	45	33	4.5847
Middle frontal gyrus_L	− 36	39	36	24	3.5186
Precuneus_L	− 9	− 48	36	18	3.3666
Cerebelum_4_5_L	− 24	− 39	− 27	24	− 3.7654
Inferior frontal gyrus (opercular part)_L	− 48	12	6	18	− 3.8408
Female vs. Male					
Middle frontal gyrus (orbital part)_L	− 21	54	− 9	23	4.1773
Superior frontal gyrus_Medial_L	− 6	54	39	18	− 3.6465
Inferior frontal gyrus (triangular part)_L	− 45	21	27	28	− 3.6841
Cerebelum_6_R	21	− 69	− 27	20	− 3.7063
Postcentral_L	− 60	− 15	39	42	− 3.7825
Middle temporal gyrus_L	− 60	− 45	0	23	− 3.7848
Inferior parietal gyrus_R	57	− 27	51	29	− 4.174
Middle frontal gyrus_R	48	42	9	19	− 4.2704
Inferior frontal gyrus (opercular part)_R*	39	9	30	40	− 4.2732
Inferior parietal gyrus_R*	39	− 39	45	36	− 4.3933
Inferior parietal gyrus_L*	− 54	− 45	48	37	− 4.5862
Cerebelum_Crus1_L*	− 15	− 78	− 27	43	− 4.9005
Inferior parietal gyrus_L*	− 33	− 51	48	51	− 5.7512
ALFF					
TN vs. control					
Supramarginal gyrus_R*	60	− 39	36	32	− 5.1827
Middle frontal gyrus_R*	39	33	33	79	− 4.8756
Supplementary motor area_L*	0	9	69	57	− 4.8042
Anterior cingulate gyri_L	− 3	33	30	27	− 4.1339
Inferior frontal gyrus (triangular part)_L*	− 36	30	24	35	− 3.7833
Postcentral gyrus_R	60	− 3	36	24	3.576
Left vs. Right affected side					
Middle occipital gyrus_L*	− 27	− 87	24	41	− 4.8634
Superior parietal gyrus_R	15	− 57	51	31	− 4.027
Middle occipital gyrus_L	− 33	− 93	12	20	− 3.9095
Cerebelum_Crus2_L	− 15	− 78	− 36	21	− 3.7302
Inferior frontal gyrus_(opercular part)_R	63	12	3	18	3.8474
Superior temporal gyrus_R*	63	− 18	9	27	4.0122
Superior temporal gyrus_L	− 60	− 21	9	29	4.0651
Middle temporal gyrus_R	57	− 63	0	18	4.237
Female vs. Male					
Inferior parietal gyrus_L*	− 51	− 51	39	18	− 4.854
Inferior parietal gyrus_R	42	− 42	54	25	− 4.1518
Superior occipital gyrus_R	15	− 87	33	25	− 3.8642
Middle temporal gyrus_L	− 63	− 45	3	27	− 3.5619
Middle temporal gyrus_L	− 48	− 18	− 15	24	3.7365
Superior frontal gyrus_R*	18	24	60	177	6.1168
High vs. low NVC scores					
Anterior cingulate gyri_R*	12	33	27	26	4.6946
Precuneus_R*	12	− 57	39	28	4.2939

*ALFF* amplitude of low frequency, *L* left, *MNI* montreal neurological institute, *NVC* neurovascular compression, *R* right, *ReHo* regional homogeneity.

*Voxel-based, independent-sample t-test with familywise error (FWE) corrections (*P* < 0.01).

^#^FWE-correction Voxels.

### Brain areas with ReHo and ALFF differences between the right and left affected side groups

**Figure 2:**
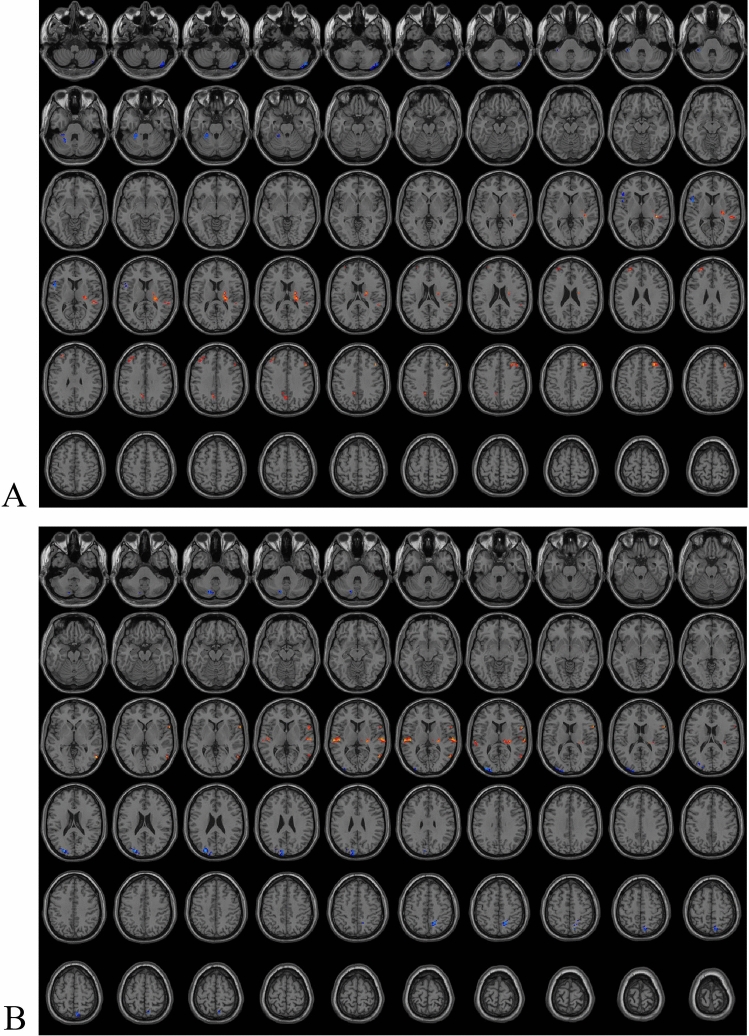
Brain areas with ReHo (**A**) and ALFF (**B**) differences between different affected sides.

Compared to TN patients with right-sided disease, the brain areas with significantly enhanced ReHo values in TN patients with left-sided disease included bilateral middle frontal gyrus and left precuneus (Fig. 2A, Table 2). Compared to TN patients with right-sided disease, the brain areas with significantly weakened ReHo values in TN patients with left-sided disease included left superior cerebellum and left inferior frontal gyrus (opercular part) (Fig. 2A, Table 2). After FWE correction, the differential in ReHo values between the two groups were mainly located in the right middle frontal gyrus (Table 2). Compared to TN patients with right-sided disease, the brain areas with significantly enhanced ALFF values in TN patients with left-sided disease included right middle temporal gyrus, right inferior frontal gyrus (opercular part), and bilateral superior temporal gyrus (Fig. 2B, Table 2). Compared to TN patients with right-sided disease, the brain areas with significantly weakened ALFF values in TN patients with left-sided disease included left inferior cerebellum, left middle occipital gyrus, and right superior parietal gyrus (Fig. 2B, Table 2). After FWE correction, the differential brain regions in ALFF values between the two groups were mainly located in the left middle occipital gyrus and right superior temporal gyrus (Table 2). Clinical analysis showed no significant differences in the NVC (5.39 ± 2.75 vs. 3.60 ± 2.82) and VAS scores (8.17 ± 0.99 vs. 8.45 ± 1.05) between the two groups (*P* > 0.05).

### Brain areas with ReHo and ALFF differences between different sexes

**Figure 3:**
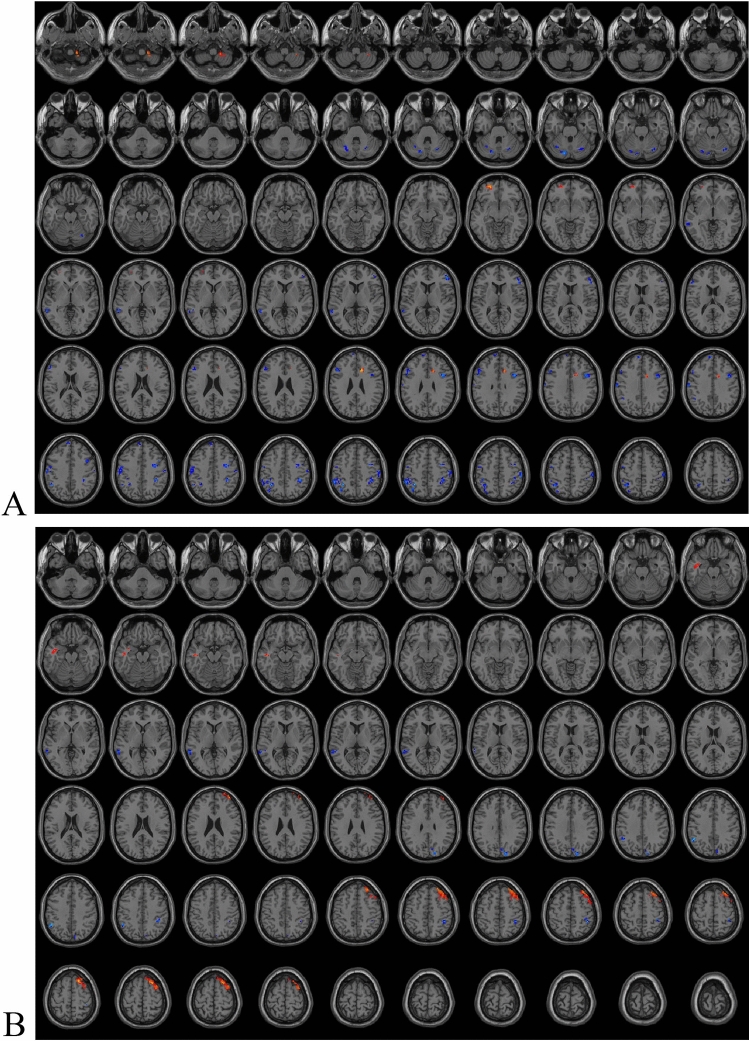
Brain areas with ReHo (**A**) and ALFF (**B**) differences between different sexes.

Compared with the male TN patients, the brain areas with significantly enhanced ReHo values in female TN patients included left middle orbital frontal gyrus (Fig. 3A, Table 2). Compared with the male TN patients, the brain regions with significantly diminished ReHo values in the female TN patients included the bilateral cerebellum, bilateral inferior parietal gyrus, several frontal gyrus, left postcentral gyrus, and left middle temporal gyrus (Fig. 3A, Table 2). After FWE correction, the differential brain regions in ReHo values between the two groups were mainly located in the bilateral inferior parietal gyrus, left superior cerebellum, and right inferior frontal gyrus (opercular part) (Table 2). Compared with male TN patients, the brain regions with significantly enhanced ALFF values in female TN patients included the right superior frontal gyrus, and left middle temporal gyrus (Fig. 3B, Table 2). Compared with the male TN patients, the brain areas with significantly diminished ALFF values in female TN patients included the right occipital superior gyrus, left middle temporal gyrus, and bilateral inferior parietal gyrus (Fig. 3B, Table 2). After FWE correction, the differential brain regions in ALFF values between the two groups were mainly located in the left inferior parietal gyrus and right superior frontal gyrus (Table 2).

### Brain areas with ReHo and ALFF differences between different NVC scores

Compared to low NVC scores, brain regions with ReHo values in patients with high NVC scores had no clear directivity. Compared to low NVC scores, brain areas with significantly enhanced ALFF values in patients with high NVC scores included the right precuneus and right anterior cingulate gyrus before and after FWE correction (Table 2).

## Discussion

TN is a unique form of peripheral neuralgia that significantly impacts the daily functioning and productivity of individuals, yet the underlying central mechanism remains incompletely understood. In the present study, a combination of ReHo and ALFF analyses were employed to investigate abnormalities in local homogeneity and spontaneous brain activity among TN patients. ALFF quantifies the magnitude of temporal fluctuations, whereas ReHo assesses the local synchronization of neighboring voxels. Although the findings from these two methods diverge, they collectively offer valuable insights into alterations in regional spontaneous brain activity in TN patients.

### DMN and the experience of pain in TN patients

#### Medial prefrontal region

The prefrontal cortex is implicated in many higher cognitive functions, including executive cognitive control and behavioral inhibition^24^. Our study revealed reduced ALFF signal in the right middle frontal gyrus and left triangular inferior frontal gyrus in TN patients compared to controls, with female TN patients also exhibiting altered ALFF signals in the right superior frontal gyrus relative to male TN patients. We hypothesize that the activation of these regions may be triggered by the electric shock-like sensations experienced in TN. Davis et al.^9^ found cortical thinning and loss of gray matter in patients with TN in the medial frontal and orbitofrontal cortices. These structural changes may underlie the decreased resting-state spontaneous activity observed in specific regions of the medial frontal lobe in this study. Activation of the prefrontal areas of the brain has been linked to pain regulation. At the same time, frequent and intense pain can disrupt the patient’s sleep experience, thus causing abnormalities in function in vivo and further causing frontal cortex dysfunction^25^. In this study, we found that compared to controls, TN patients had significant inhibition of ReHo signaling in the middle frontal gyrus bilaterally, while there was enhanced activation in the right supraorbital frontal gyrus. These findings suggest that the experience of pain and its associated functional limitations may contribute to aberrant neural functioning within frontal lobe-related regions. The enhanced brain activation observed in the right supraorbital frontal gyrus may represent a compensatory mechanism in response to these alterations. Likewise, individuals with left-sided TN exhibited increased activation in the right middle frontal gyrus relative to those with right-sided TN. When examining sex differences, female patients with TN displayed notable changes in brain activity within various regions, particularly in the right prefrontal areas, compared to male TN patients, which potentially implicating emotional control. The modifications in the medial prefrontal cortex induced by pain additionally impacted emotional regulation in females.

#### Parietal region

The inferior parietal lobule, comprising the supramarginal gyrus and angular gyrus, with intricate fibrous connections, is intricately linked to language function. Our study revealed that female TN patients exhibited decreased ALFF and ReHo signals in the inferior parietal lobule compared to male TN patients. This structure serves as a functional area for sensory stimulation and cognition, and females show a more robust metabolism, especially in language signal processing. The supramarginal gyrus also is also associated with emotional recognition and social interaction^26,27^. The precuneus is a region of heightened metabolic activity within the DMN that plays a crucial role in various higher cognitive functions and exhibits extensive functional connectivity with the frontal, parietal, temporal, and occipital cortex^28,29^. The precuneus is closely related to self-perception and emotional processing. In patients with TN, individuals with left-sided disease demonstrated activation of the left precuneus compared to those with right-sided disease. It can be hypothesized that frequent discharge-like stimulation in TN patients with left-sided disease caused a compensatory mechanism in the precuneus. Previous studies of TN have similarly found abnormal ReHo in the middle temporal gyrus and precuneus on brain scans^30^. Alterations in the precuneus and cingulate region were mainly seen in comparing patients with different degrees of NVC. Therefore, the severity of NVC remains an essential factor in judging the seriousness of TN.

### Temporal lobe region and the experience of pain in TN patients

The frontotemporal lobe, the most advanced brain region, is a major component of the DMN, and is also the most structurally and functionally vulnerable region^31^. The anterior temporal lobe is generally associated with emotional and mental activity. In the present study, there was a significant inhibitory signal in the middle temporal gyrus in TN patients compared to controls, which, combined with data from the frontal lobe, suggests that mood and emotion regulation remain vital factors influencing these two brain regions. The supramarginal gyrus is a crucial component of the auditory speech center, mainly involved in auditory processing and sound information in concert with the angular gyrus^32,33^. Comparing different sexes, we found that female patients had lower signals in the inferior parietal marginal angular gyrus, which, combined with the fact that TN disease is characterized by a decline in hearing with the corners of the mouth as trigger points, could explain the results of abnormalities in brain regions related to auditory language processing.

### Cerebellum and the experience of pain in TN patients

The cerebellum receives a complex source of afferent fibers, including those from the prefrontal cortex, parietal cortex, temporal cortex, and sensory-motor cortex. It also gets a wide range of somatosensory input via the spinal cerebellar pathway. The present study showed that the bilateral cerebellar lobe area II was significantly activated in TN patients. Areas I and II of the cerebellum are connected to the dorsolateral prefrontal and parietal cortex and are responsible for the association with the brain, so their activation may be related to the increased long-term and high frequency of sensory input in TN. In addition, male TN patients showed activation of left cerebellar superior regions. The activation may be related to the different pain thresholds in men and women. But, it cannot be ruled out that the activation is associated with psycho-emotional processing.

### Occipital lobe and the experience of pain in TN patients

The occipital lobe is comprised of mostly visual cortex^34^ and is a visual processing center responsible for visual communication^35^. In a study in rats, the occipital lobe has recently been shown to be involved in the downstream inhibitory mechanisms of pain^36^. Studies on diabetic neuropathic pain (DNP) have also found a negative correlation between cortical network activity in this region and the intensity of DNP^37^. In this study we found that TN patients with right-sided disease had higher ALFF signals in the left middle occipital gyrus, which may be related to the activation of analgesic inhibitoury mechanisms. Although there is no evidence to elaborate on the mechanism of the role of the middle occipital gyrus in the aggregation and processing of visual information, abnormalities in the middle occipital gyrus have been found in several psychiatric disorders^38–40^. Therefore, the prolonged intrusion of TN may affect the patient’s mood and mental regulation, which in turn causes abnormalities in the metabolism of the middle occipital gyrus. Previous studies have demonstrated abnormal occipital lobe activation in patients with facial spasms^41^. Given the similarities between facial spasms and TN, we suspect that persistent eye socket pain may affect visual pathways, especially in those patients with TN involving V1 and characterized by orbital pain.

### Sensory-motor cortex and the experience of pain in TN patients

The precentral gyrus (primary motor cortex) and the supplementary motor area are essential parts of the sensory-motor cortex and are closely related to somatic movements. In TN patients, even simple nonpainful actions can cause painful episodes, which are relieved if facial movements are restricted. Numerous studies have shown that the precentral gyrus can produce sensory pain response to repetitive TN, maxillary motor inhibition, and facial muscle tension^42^. The somatosensory network in healthy adults primarily encompasses bilateral precentral gyrus, postcentral gyrus, middle frontal gyrus, middle occipital gyrus, insula, and cuneus^43^. The postcentral gyrus serves as the central hub of this system, playing a crucial role in the integration of somatosensory and motor information^44^, as well as in emotion regulation^45^. Research indicated that individuals with depression exhibited notably reduced ALFF values in the postcentral gyrus compared to their healthy counterparts^46^. In the present study, TN patients showed significant activation of the right postcentral gyrus and significant inhibition of left supplementary motor area compared to controls. Enhanced functional activity in the posterior central gyrus is hypothesised to be related to compensatory mechanisms for motor deficits. In contrast, the low signal presented in the supplementary motor area may result from maladaptation that occurs in the process of regulating motor function deficit. The activation of the postcentral gyrus region in TN patients demonstrates that the degree and frequency of nociceptive episodes and the patient’s expectations of pain influence alterations in the sensory cortex to a greater extent than the restriction of facial movements.

## Future directions and study limitations

Our findings should be interpreted in light of several limitations. The sample size of TN patients used in this study was limited, and some influencing factors may be confounded in subgroup analyses. For example, in the gender-subgroup analysis, since TN is highly prevalent in females, and female cases in our study far exceeded those of males, the results can only show some trends. Since the patients recruited in our study did not emphasize the initial onset, most of them had a history of taking medication. It is not clear whether the medication has some effect on brain function. TN is a chronic disease and the effect of the duration of the disease on brain function is also not known. At the same time, TN is a recurrent disease with great individual variation in inter-episode intervals and frequency of episodes, and precipitating factors, so how to calculate the course of the disease has been a controversial issue. There is no uniform standard whether the time of onset of disease should be calculated from the time of first onset or from the time of current onset. Previous studies have also shown this limitation. This is also the main reason why the disease course factor was not cited in the investigation of influencing factors in this study. Another reason is that the sample size of this study is small, which limits the grouping of TN patients by disease course. Therefore the conclusions drawn from this study need to be supported by more dimensional evidence. It is necessary to expand the sample size in future studies, especially focusing on the effects of disease duration, medications, or other treatments on brain function. In future studies, it is also necessary to refine the brain region localization of TN patients, and on this basis, further analyze the functional connectivity and topological properties of the brain, and compare the data with other peripheral neuralgia or other neurovascular compressive disorders to make the conclusions more convincing.

## Conclusion

In summary, the changes in ReHo in TN patients suggest that with the functional impairment of the central pain area, the functional areas controlling memory and emotion also change during the progression of the disease. Decreased ALFF in default networks and limbic systems, as well as increased ALFF in sensory and perceptual brain regions, may reflect the abnormal influence of the continuous repetitive sensory input of TN on brain network function. TN patients with different sexes, affected sides, or degrees of nerve damage may have different central mechanisms. These results provide us with new ideas to understand and analyze the central mechanisms of TN and provides a foundation for future research.

## Data Availability

The data sets used and analyzed in this study are available by the corresponding authors upon reasonable request. The data are not publicly available due to privacy or ethical restrictions.
